# Scale-up integrated care for diabetes and hypertension in Cambodia, Slovenia and Belgium (SCUBY): a study design for a quasi-experimental multiple case study

**DOI:** 10.1080/16549716.2020.1824382

**Published:** 2020-10-14

**Authors:** Josefien van Olmen, Sonia Menon, Antonija Poplas Susič, Por Ir, Kerstin Klipstein-Grobusch, Edwin Wouters, José L. Peñalvo, Črt Zavrnik, Vannarath Te, Monika Martens, Katrien Danhieux, Savina Chham, Natasa Stojnić, Veerle Buffel, Sokunthea Yem, Gareth White, Daniel Boateng, Zalika Klemenc-Ketis, Valentina Rupel Prevolnik, Roy Remmen, Wim Van Damme

**Affiliations:** a Department of Public Health, Institute of Tropical Medicine Antwerp, Antwerp, Belgium; b Department of Sociology, University of Antwerp, Antwerp, Belgium; c Community Health Center Ljubljana, Slovenia; d Department of Family Medicine, Faculty of Medicine, University of Ljubljana, Ljubljana, Slovenia; e National Institute of Public Health, Ljubljana, Cambodia; f Julius Global Health, Julius Center for Health Sciences and Primary Care, University Medical Center Utrecht, Utrecht University, Utrecht, The Netherlands; g Division of Epidemiology and Biostatistics, School of Public Health, Faculty of Health Sciences, University of the Witwatersrand, Johannesburg, South Africa; h Department of Family Medicine, Faculty of Medicine, University of Maribor, Maribor, Slovenia

**Keywords:** Type 2 diabetes, cardiovascular disease, implementation research, quasi-experimental design, chronic care

## Abstract

Health systems worldwide struggle to manage the growing burden of type 2 diabetes and hypertension. Many patients receive suboptimal care, especially those most vulnerable. An evidence-based Integrated Care Package (ICP) with primary care-based diagnosis, treatment, education and self-management support and collaboration, leads to better health outcomes, but there is little knowledge of how to scale-up. The Scale-up integrated care for diabetes and hypertension project (SCUBY) aims to address this problem by roadmaps for scaling-up ICP in different types of health systems: a developing health system in a lower middle-income country (Cambodia); a centrally steered health system in a high-income country (Slovenia); and a publicly funded highly privatised health-care health system in a high-income country (Belgium). In a quasi-experimental multi-case design, country-specific scale-up strategies are developed, implemented and evaluated. A three-dimensional framework assesses scale-up along three axes: (1) increase in population coverage; (2) expansion of the ICP package; and (3) integration into the health system. The study includes a formative, intervention and evaluation phase. The intervention entails the development and implementation of an improved scale-up strategy through a roadmap with a minimum dataset to monitor proximal and distal outcomes. The SCUBY project is expected to result in three different roadmaps, tailored to the specific health system and country context, to progress scale-up of the ICP along three dimensions. These roadmaps can be adapted to other health systems with similar typology. Implementation is expected to increase the number of well-controlled patients with type 2 diabetes and hypertension in Cambodia, to reduce inequities in care and increase patient empowerment in Belgium and Slovenia.

## Background

Globally the burden of Non-Communicable Diseases (NCDs) constitutes a major public health concern. The prevalence of type 2 diabetes (T2D) continues to increase worldwide, a trend attributed to ageing, rapid urbanisation, and obesogenic environments [1], particularly in lower-income populations [2]. According to 2019 global estimates, 463 million adults live with T2D and 1.13 billion people live with hypertension (HT) [3,4]. HT is an important global health challenge due to its high prevalence and resulting risk of developing chronic kidney and cardiovascular diseases [5]. Due to shared risk factors, patients with T2D are also at higher risk of HT [6]. This increasing burden is a challenge for health systems worldwide. Suboptimal responses result in a large proportion of T2D and HT patients, early development of complications and high cost. Comorbidity of T2D and HT calls for comprehensive patient-centred care [7]. Effective interventions for treatment and control of both conditions are available and cost-effective [7,8] and include the following overall elements: (a) early detection and diagnosis, (b) treatment in primary care services, (c) health education, (d) self-management support to patients and caregivers, and (e) collaboration between caregivers. These bundled interventions can be identified as an ‘integrated care package’ (ICP). They are in line with chronic care models and WHO guidelines on integrated care and essential interventions for diabetes and hypertension [9–11]. Further, there is strong evidence that this ICP, when implemented, leads to improved care processes and responsiveness of health care to patients’ needs and to better health outcomes [12].

However, large parts of the world’s population lack access to this ICP. Health systems in low- and middle-income countries (LMICs) do not include the ICP elements in their essential primary care services, because of limited resources, competing priorities and insufficient human capacity. In high-income countries (HICs), vulnerable groups such as elderly, with comorbid conditions and people from lower socio-economic strata often do not receive appropriate care and support [13]. There is a lack of knowledge on how to implement ICP into existing health systems. Intervention studies on integrated care for HT and T2D provide little information on implementation [14]. This hinders replication in other settings and the development of scale-up strategies.

The SCUBY project is a large-scale quasi-experimental multi-country research project addressing this implementation research gap. Three countries have been purposively selected based on their health system characteristics and different stages in scale-up: Cambodia, Slovenia and Belgium. Each country currently develops a strategy for scale-up of the ICP for T2D and HT tailored to their burden of T2D an HT, current ICP implementation, health system decentralisation and budget allocation. This innovative case selection combined with a robust evaluation enables to study the development, implementation and effectiveness of scale-up strategies for integrated care for T2D and HT in different types of health systems.

## Aims and objectives

The aim of the SCUBY project is to provide evidence on the scaling up of the ICP for T2D and HT for dissimilar types of health systems, through the development and evaluation of roadmap-strategies that can be adapted to be used in different contexts.

The specific research objectives of SCUBY are to: (1) analyse the organisational capacity to scale-up the ICP for T2D and HT in Cambodia, Slovenia, and Belgium and to assess their respective contextual barriers and facilitators (2) develop and implement roadmaps for a national scale-up strategy in each country; (3) evaluate the impact on health outcomes, coverage, and quality of care through the scale-up of the ICP; and (4) generate lessons across contexts on the scale-up strategies for integrated care for T2D and HT.

## Study design

The project has a quasi-experimental multiple case study design. Each country is a case of scale-up of the ICP for T2D and HT. The project commences with a formative phase (year 1) followed by an intervention phase (years 2–3) and an evaluation phase (year 4). The multi-case analysis will be drawn at different moments and at different levels, following the reciprocal learning approach [15].

During the formative phase, the focus will be on the ICP package and assessment of the current implementation and barriers and facilitators in each country at three levels (micro-meso-macro or individual-organizational-national). The intervention phase will entail the development of a roadmap in collaboration with implementation stakeholders. As the nature of the intervention does not allow for controlled exposure, a quasi-experimental study design will be used for a before and after evaluation of proximal and distal outcomes [16].

### Study setting

**Cambodia** is a lower-middle-income country that has approximately 15 million people and an annual health expenditure of 79.6 USD per capita in 2016 [17]. It has a public health system with strong support from government and donor organisations and a rapidly growing private sector. Cambodia is currently undergoing an epidemiological transition with emerging prominence of NCDs. T2D and HT are the most common NCDs with a prevalence between 5 and 10% for T2D and 11% for HT in the general adult population [18]. The mean annual expenditure on diabetes per person was 52.7 USD in 2010 [19]. The Ministry of Health has identified all components of the ICP as important and relevant for Cambodia and is therefore committed to implementing ICP through the WHO Package of Essential Noncommunicable (PEN) disease Interventions in each operational health district (OD) [11]. In the current situation, there are three dominant variations of how ICP is delivered within an OD: a) ODs with a hospital-based diabetes clinic only; b) ODs with a diabetes clinic and health centres that perform PEN-identified tasks; c) ODs with community-based patient support collaborating with the district hospital. In some ODs b and c are combined.

**Slovenia** has 2 million inhabitants and an annual health expenditure of 2263 US dollars per capita in 2015 [20]. The health system is to a large extent, financed from national health insurance and has mixed public-private providers. The national prevalence of T2D is 5.1% and the mean annual expenditure on diabetes was 2608 USD per person per year in 2016. Since 2011, the government has invested in the scale-up of upgrading family care practices for chronic diseases management through a ‘model practice’ [21]. Protocols for management of patients with T2D, HT and other chronic diseases were implemented and monitored through quality indicators. This has standardized diagnosis, treatment, health education and referral for patients. Community nurses were deployed to reach vulnerable patients.

**Belgium** has 11 million inhabitants and a health expenditure of 4507 USD per capita in 2017 [22,23]. It has a privatized health-care system, funded through a mix of direct government payment and refunding of patients through third-party payers. Health-care providers and patients enjoy a high degree of autonomy of choice. In 2017, an estimated 6.1% of people had diabetes and the mean annual expenditure on diabetes was 6612 USD [19]. Many diabetes patients have other comorbidities, mostly hypertension [24]. 30% of patients with chronic conditions, especially elderly people belonging to vulnerable groups such as having comorbidities of socio-economic problems, express the need for additional support [25]. Since 2009, the government has restructured chronic care for diabetes patients, differentiating roles for primary and secondary care and for self-management support, through care pathways. Multiple projects have been developed to better reach these vulnerable groups and to reduce fragmentation in the system through local health-care networks. These projects implement ICP through primary care practices, which vary in their organisational model: a) monodisciplinary general practice; b) multidisciplinary health centre with support from health educator or dietician; c) a multidisciplinary health centre with a capitation payment system in which patients subscribe and the centre gets paid fixed fee.

### Study population

This study prioritises vulnerable populations. In Cambodia, all people with T2D and HT are considered vulnerable, and therefore, the scale-up targets the whole population using the public health services. In Slovenia and Belgium, the scale-up focuses on vulnerable groups, being defined as elderly patients (above 65) and/or patients with chronic comorbidities. Inclusion criteria include belonging to the target population, there are no additional exclusion criteria.

### Scale-up framework and intervention

**Figure 1.**
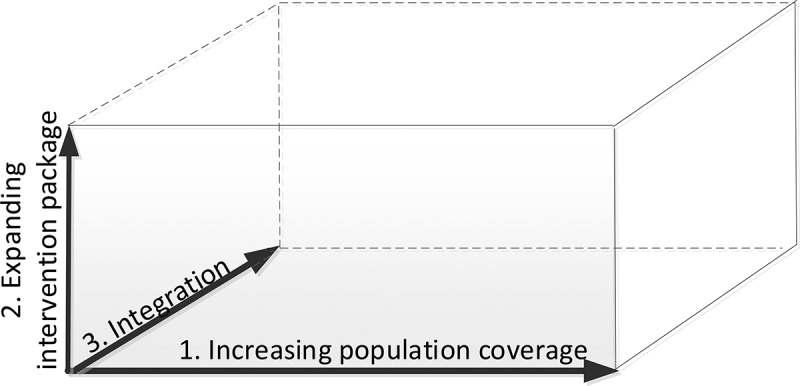
The three-dimensional scale-up framework to conceptualise scale-up as 1) increasing population coverage; 2) expanding the intervention programme; and 3) integration into health system and services (based upon Meessen et al [27], inspired by the universal coverage framework [28]).

Scale-up is the ‘efforts to increase the impact of health interventions so as to benefit more people and to foster policy and programme development on a sustainable basis’ [26], by means of the implementation of an evidence-informed country-specific roadmap. We have developed a three-dimensional framework of scale-up of the ICP: (1) increasing population coverage; (2) expanding the intervention package; and (3) integration of the ICP into the health system (Figure 1).

A scale-up strategy refers to the processes and actions by which the ICP is brought to scale. The scale-up literature categorises scale-up strategies according to the degree of the intention of scale-up, formal planning and locus of initiative in three types [26]: a) top-down strategies whereby the central level decides to implement the innovation and institutionalises it through planning, policy changes or legal action; b) horizontal strategies to expand geographically or population-based; and c) diversification strategies referring to adding new elements to an existing intervention. The three countries follow this categorisation in the current focus and approach: a government steered top-down (type a) strategy in Cambodia, a horizontal strategy (type b) in Belgium, and a diversification scale-up strategy (type c) in Slovenia. The SCUBY intervention is a roadmap that adapts the scale-up strategy to include new activities and strategies from the other categories. It contains targets, planning and monitoring of scale-up strategies, identifying actors, actions and timelines. The scale up roadmaps will be based upon the formative findings and adapted in cyclical improvement process, with stakeholders through policy dialogues. SCUBY will produce three roadmaps adapted to specific health system and context.

## Methods

This paper describes the overall design and methods for each project phase. Table 1 contains an overview of research questions (RQs), key variables and information, measurement instruments and data collection methods for the studies in each phase.

**Table 1.** Variables and key information, measurements and implementation instruments and data collection method in the SCUBY project.

	RQ	Variables and key information	Measurement/implementation instruments	Data collection method
	**Formative phase**			
	**Macro-level: Context analysis**			
1a	What is the current strategy, capacity and engagement of stakeholders to scale-up the ICP and what is the societal and health system context that are potential barriers or facilitators to scale-up?	Current strategy and plans to scale-up ICP; national context/health system barriers and facilitators	Study specific semi-structured interview guide	Indepth interview and document analysis
1b	Who are the key stakeholders in the scale-up?	Stakeholder mapping and power and interest analysis	Guidelines for Conducting a Stakeholder Analysis	Indepth interview and document analysis
1c	What is the financing system for ICP?	Financing system for ICP	Topic guide on health and diabetes financing	Key informant interviews, policy and administraion documents, related studies national health accounty
	**Meso-level: Analysis of current ICP implementation and cost**	**Analysis of current ICP implementation and cost**		
1d	What is the present implementation of the ICP and (variation in) organisational models in the current pilot sites	Implementation of the ICP; organisational models and organisation context	ICP implementation assessment grid; focus group discussion guide	researcher observation and interviews in selected pilot sites
1e	What are the costs of implementing the ICP from the health system and health provider perspective	Provider-side costs of ICP	Study specific costing tool	national health accounts and health insurance data (Slov/Bel), existing lit, rapid health facility survey (Cam)
	**Micro-level: individual outcomes**	**Outcomes at individual level**		
1f	What are the outcomes of the ICP as currently implemented	Number of people with T2D/HTN; Proportion of people tested/diagnosed/retained in care/on treatment/followed-up/well controlled	Cascades of care, starting from prevalence in reference year.	household survey (Cam), combination of databases from insurance, laboratories and primary care networks (Slo,Bel)
1g	What is the cost for the patient and what are barriers to care?	Patient side barriers to care; patient out of pocket expenditure	Study specific demand side costing tool	qualitative through focus group and indepth interview with patients; quantitative through patient diaries for sample of patients through population based survey
	**Intervention phase: scale-up strategy and implementation**			
	**Roadmaps**	**Scale-up strategy**		
2a	How can we, in co-creation with stakeholders, optimize the current Scale-Up (S-U) strategy	Recommendations for improved scale up	Policy dialogues and roadmaps	Formative findings study 1–3
2b	Which mechanisms can be identified for the relationship between scale-up strategy, actors and context?	Empirical and theoretical evidence	Scientific enquiry	dialogue practice and theory, implementors and researchers
2c	What is the minimum indicator set to monitor	minimum monitoring data set	quantitative data on CoC; qualitative data on process, barriers and context	survey statistics extracted from routine data; observations, key information interviews, patient interviews, project documents
2d	What are the projected cost for different scenarios	projected cost	costing models	Formative findings study 1–3
	**Evaluation phase: outcome and impact evaluation**			
3a	How has the roadmap been implemented and to what extent, and how is the context influencing the implementation of the scale-up strategies, including cost	implementation of the roadmap	Reach (Number of scaleable units covered by the scale-up); Acceptability (measured by Affective Attitude, Burden, perceived effectiveness, Opportunity Costs, Intervention Coherence, implementors’ Self-efficacy, and Ethicality) and feasibility of the scale-up strategy (measured by adaptation and fidelity of implementation)	project diaries, interviews with implementors and key informant interviews
3b	What is the progress on each of the three axes of the scale-up box	progress on (a) the population coverage(b) the expansion of the ICP (c) integration	(a) reach; target population living in area; number of people actually covered by intervention (b) number of components added to the ICP (c1 organisation level, ICP implementation grid see 2) normalisation process theory (c2health system level) sustainable financing arrangements for the ICP, provider payment mechanisms stimulate health education and self-management, human resource planning for teamwork in facilities and with community, care pathways, common monitoring	Quantitative data through routine data or population survey. Qualitative data at endline through project diaries, interviews with implementors, and key informant interviews and practice
3c	What is the impact of the scale-up on the control of T2D and HT ?	impact of the scale-up on the control of T2D and HT	CoC (see 3)	Interrupted timeseries from routine data
3d	What are the costs of the scaled-up ICP, for the health system and for the patient?	costs of the scaled-up ICP: (a) health system and (p) patient perspectove	(a) cost of human resources and service delivery (b) out of pocket expenditure (see 3)	(a) primary qualitative and quantitative data (b) patient survey

### Formative phase

The formative phase aims to understand the current degree of implementation of ICP and its effects, the current scale-up strategy and main actors, and the barriers and facilitators for scale-up. This phase entails three levels of analysis for a comprehensive assessment. The project uses a concurrent mixed methods approach, in which quantitative analyses are performed to assess individual-level outcomes and costs, and qualitative analyses to examine perceptions, context and processes.

#### Context analysis (macro-level)

These analyses pertain to the health system and national context, answering the following RQs: What is the current strategy to scale-up the ICP and what are national and health system barriers or facilitators? (RQ1a); Who are the key stakeholders, what is their capacity and level of engagement? (RQ1b); What is the financing system for ICP? (RQ1c).

For RQ1a and RQ1b, a stakeholder analysis will be carried out to identify and map stakeholders’ role, interest and vision of the current state of implementation and plans for scale-up, also identifying key stakeholders for the intervention phase in each country. Potential participants were identified using desk research, networking and snowball sampling. Examples of potential stakeholders include the Department of Preventive Medicine of the Ministry of Health and the WHO Country Office in Cambodia; professional and patient organisations and the Health Insurance Institute in Slovenia; and pilot project leaders, reform implementors, and federal and regional authorities in Belgium. Data collection will be carried out with in-depth interviews (webannex 1, based upon WHO stakeholder analysis guide [29] and the ExpandNet/WHO framework outlining five strategic choice areas for scale-up [26]) and document analysis. The analysis will be partly deductive (based upon earlier mentioned frameworks) and partly inductive (based upon emerging themes from the interviews). For RQ1c, the WHO tool for financial system analysis [30] will be used to assess the economic context, revenue collection, pooling and allocation, and remuneration systems and incentives for providers and patient, in particular, ICP for T2D and HT [31]. Data will be collected via key informant interviews, document analyses and national health account analysis.

#### Analysis of current ICP implementation and costs (meso-level)

This organizational analysis comprises the following RQs: What is the present implementation of the ICP and (variation in) organisational models in the current pilot sites (RQ1d)? What are the costs of implementing the ICP from the health system and health provider perspective (RQ1e)?

#### Sampling

We will purposely select areas where the ICP is currently implemented, and in those areas, we aim for maximum variation of the three different organisational models in Belgium and in Cambodia (see study settings). Within these areas, we will select ‘units of analysis’ aiming for an optimal mix using random sampling of each of the organisational models. In Slovenia with only one organisational model, one rural and one urban area will be selected to capture variability, and within those areas one representative unit will be selected. In Belgium, two urban areas and one rural area will be selected from which 10 practices of each organisational model will be randomly selected. In Cambodia, five ODs will be selected with respective model a, b, c, and combined b and c (see context). If the organisational model includes health centres (model b and c), three health centres in the OD will be chosen randomly, together with the diabetes clinic. A sampling frame is provided in a webannex 2.

#### Data collection

For RQ1d, the ‘ICP Implementation assessment framework’ (webannex 3) will be used. It draws upon two validated and widely used instruments to assess integrated chronic care, the assessment of chronic illness care tool for the six domains of the chronic care framework [32] and the innovative care for chronic conditions situation assessment for the implementation of strategic steps in the health-care organisation [33]. Document analysis, practice observations and in-depth interviews with health facility managers and key informants will be performed, as well as focus group discussions (FGD) with patients, medical doctors, nurses and other relevant health workers or community-based actors, on obstacles and facilitators at meso and micro-level. Inclusion criteria for patients relate to the pre-identified vulnerability criteria. To broaden the perspective on vulnerability taking into account other dimensions such as socio-economic, our sampling strategy will also aim to include people with such characteristics, and collect data on these dimensions. The scoring instrument will be pilot-tested and adapted to the contexts of the three countries, adapting language and generic concepts to the national delivery models. Two researchers will independently complete grading and come to a consensus score, triangulating data with multiple sources. Furthermore, a generic topic guide which will be adapted for different groups, contextualised and translated for each country (webannex 4). The quantitative score will provide an indicator for the depth and width of ICP implementation and the qualitative analysis will provide understanding of the organisational context. For RQ1e, cost will be estimated for a one-year time frame. Data collection will start from a review of publications and reports. In Cambodia, a rapid facility-based survey will be performed (webannex 5). In Belgium and Slovenia, information will be retrieved for secondary data analysis of existing health financing/accounting systems and reports, complemented with primary data collection through FGD with health-care providers, key information interviews and financial record systems. The analysis will assess total costs and, where possible, the costs per unit, units being: cost per facility/provider; total annual cost by ICP component; total cost by cost categories.

#### Outcomes at patient level (micro-level)

This level of assessment includes the following RQs: What are the outcomes of the ICP as currently implemented (RQ1f)? What is the cost for the patient and what are barriers to care (RQ1g)? For RQ1f, a Cascade-of-Care (CoC) approach is developed assessing outcomes for T2D and HT across the care continuum [34]. Two generic CoCs – one for T2D and one for HT – will be constructed for each country. The CoC consists of 6 bars: 1) Number of people with T2D or HT measured by prevalence of year x; 2) Proportion of people tested for T2D/HT, measured by number of people tested the last 3 years (x-3, x-2, x-1) (glucose or Hb1Ac blood test/blood pressure measurement); 3) Proportion of people diagnosed in year x-1 (self-report/professional-report or proxy indicator); 4) Proportion of people retained in care in year x (at least one visit at health provider); 5) Proportion of people being on treatment in year x (at least one HbA1C measurement/taking medication); and 6) Proportion of people with good T2D/HT outcomes in year x (HbA1c <53 mmol/mol; blood pressure <140/90). Since cholesterol is a common comorbid risk factor for cardiovascular disease among people with hypertension and an internationally accepted quality indicator for cardiovascular risk prevention – the context in which hypertension is often addressed -, an additional bar is added to the CoC for this population: 5b) Proportion of people who had cholesterol examination. The CoC will be based upon a single population approach for each country thus each bar will be derived from data from the same population. If not all data can be derived from the same database, we will use a combination data sources or estimations based on the existing literature. In Slovenia and Belgium, data collection and analysis will be based on aggregated data, with the population as target group. In Cambodia, primary data collection will be carried out through a household survey among the catchment population of the health facilities in the five ODs selected in step 2, through a multistage stratified random cluster sampling with a probability proportional to the OD population size. In all three countries, indicators on vulnerability will be collected on individual level, to allow stratification for these. For RQ1g, a questionnaire is developed focusing on household out-of-pocket health expenditures, including direct medical and non-medical cost and indirect cost (webannex 6). Data will be collected through the household survey (Cambodia), through in-depth interviews with the purposively selected people from the target group (vulnerable population) in Belgium, and through a survey among 200 patients with T2D and 200 with HT in Slovenia, using a proportional random selection of patients from a facility-based listing of patients with T2D and/or HT in an urban and a rural area.

### Intervention phase

#### Development and implementation of scale-up strategies

Our formative findings will prompt the following RQ for the scale-up phase: How can the current scale-up strategies be optimized and complemented, in co-creation with stakeholders (RQ2a)? Which mechanisms can be identified explaining the relationship between scale-up strategy, actors and context? (RQ2b) What is the minimum data set to monitor implementation of the ICP (RQ2c)? What are the projected costs for different scenarios (RQ2d)?

**Figure 2.**
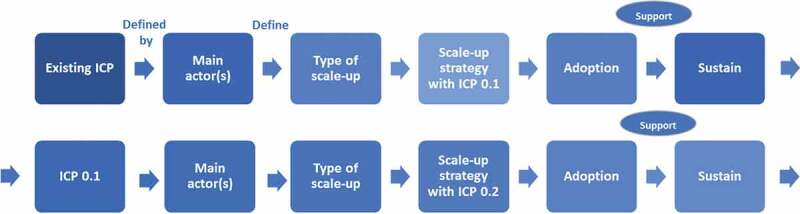
Interrelatedness between actors, context, and the intervention (roadmap actions).

A resource team of key stakeholders and organisations in each country will lead the roadmap development and implementation. These key stakeholders will be identified during the stakeholder analysis in the formative phase. The research team will support the resource team through technical advice, providing evidence and monitoring. Methods include policy dialogues and scale-up roadmaps, and theorising using the scientific circle of enquiry [35]. Policy dialogues are an approach in the policy-making process to engage with key stakeholders and to develop the country scale-up roadmaps. They will comprise structured formal events, one-to-one interactions with key stakeholders, workshops, consultations and joining ongoing dialogues within the context [36]. A scale-up roadmap is a sequential visualisation of target, planning and progression of scale-up strategies, identifying actors, actions and timelines based upon priorities in place and time. To systematically document the elements, a roadmap format will be used, inspired by two strategic scale-up frameworks in the domain of implementation science [26,37] (Figure 2). Roadmap actions (the intervention) can be re-organisation of care processes, capacity building, dissemination, advocacy and stakeholder engagement, changes in financing and monitoring. The scale-up roadmaps will be developed and adapted in an iterative improvement process. SCUBY will produce three roadmaps adapted to specific health system and context.

The research team will also use empirical findings in this stage to refine theory on scale-up and to unravel the interrelatedness between actors, context and roadmap actions (RQ2b) (Figure 2). This theorising approach implies a dialogue between the empirical process and the theoretical knowledge [35]. For RQ2c, the minimum data set will be established by extraction of routine data and publicly available surveys to the extent possible, in a time series with at least 2 points. The CoC will be the starting point for selecting indicators for the minimum dataset, but the final set of indicators will the validity, relevance and feasibility in the country context and will thus be decided with the resource team responsible for the scale-up. Qualitative data will be collected through observations in scale-up areas, interviews with stakeholders in user organisations, key information interviews, patient interviews and document analysis. For RQ2d, costing models will be built based on the collected data and possible scaling up scenarios. *We will develop three scenarios to model future cost: an optimistic scenario (all conditions fulfilled); a moderate scenario (some conditions fulfilled, moderated to delayed implementation); and a status quo scenario (business-as usual, minimal change)*.

### Evaluation phase

#### Process and impact evaluation

In this phase, the four RQs are: How has the roadmap been implemented (RQ3a)? What is the effect on the proximal outcomes, namely the progress on the three axes of the scale-up box (RQ3b)? What is the effect on the distal outcomes, namely the impact on control of T2D and HT (RQ3c)? What are the costs of the scaled-up ICP, for the health system and for the patient (RQ3d)?

For RQ3a, the implementation fidelity framework [38] is used, assessing the following aspects: Reach (Number of scalable units covered by the scale-up); Acceptability (measured by Affective Attitude, Burden, perceived effectiveness, Opportunity Costs, Intervention Coherence, implementors’ Self-efficacy, and Ethicality) and feasibility of the scale-up strategy (measured by adaptation and fidelity of implementation). For RQ3b, the progress on the three dimensions of the scale-up will be assessed through a before-after comparison: (a) the population coverage, measured by reach, and by number of people actually covered by intervention; (b) the expansion of the intervention package towards the ICP (measured through ICP implementation assessment); and (c) the integration of the ICP at the operational level will be assessed through the normalisation process theory [39] and the integration at the system level assessed by the presence of sustainable financing arrangements for the ICP, of human resource strategies for teamwork, implementation of care pathways for T2D and HT and options for shared health information systems [40]. Quantitative data will be collected through routine data (Slovenia, Belgium; estimated extraction of sample of 15 300 patients in Slovenia and 14 500 people in Belgium) or a population survey (Cambodia, 5000 people), based upon the minimum dataset (RQ2c). Qualitative data will be collected 2 years after the start of roadmap development through project diaries, interviews with implementors, and key informant interviews and practice observations. Qualitative analysis is deductive, based upon the frameworks mentioned above. For RQ3c, the CoC indicators will be used (primary outcomes), and the time series collected during monitoring of scale-up is the basis for impact evaluation. Interrupted timeseries with at least 2 (before-after implementation of scale-up roadmap) and preferably more measurements will be collected (planned first data extraction/collection in June 2020 – last in June 2022). Difference in time of follow-up will be accounted for in the analyses. The CoC data will be stratified for pre-identified vulnerability criteria (>65 years, presence of comorbidities) and for potential other dimensions of vulnerability, such as low socio-economic status, gender (unknown direction). For RQ3d, data will be collected on the cost, the human resources, and service delivery arrangements of the scale-up actions (secondary outcomes), through primary qualitative and quantitative data. After 1 year of implementing the scale-up roadmap, cost will be calculated from providers’ perspective, based on cost data routinely collected by health facilities and related institutions. For patient perspective cost, a patient survey will be repeated.

## Discussion

The SCUBY study provides a state-of-the-art research framework and innovation project in the growing domain of implementation research. It will generate knowledge on both processes and effectiveness of scale up of control and treatment strategies for two major chronic diseases in three different health system contexts. The roadmaps developed for a comprehensive scale-up (increase population coverage, expansion of the intervention package, and integration) for three types of health system contexts are expected to innovate care especially for the vulnerable subpopulations. They may also be adopted for use in other similar health systems.

The theorising approach applied in the scale-up phase will strengthen and refine existing scale-up theories with the empirical evidence collected, and enhance the knowledge on mechanisms of implementation and specifically on the science of scale-up [35]. The linking of multiple population-based data sets on health-care outcomes with meso-level data on care organisation (structure) and costs allows evidence-informed decision-making about health-care reforms.

### Strengths and limitations

The strengths of the study relate to the choice of a quasi-experimental multiple case study design; the development of cross-country theoretical frameworks and data collection tools allowing for contextual adaptation; the participatory intervention development and the comprehensive process and outcome evaluation. The selection of three cases with a different health system and contextual profile allows for lessons for diversified contexts. The risks and limitations of the study relate to the implementation. Scale-up is a complex process depending on many factors that are partly beyond the control of the resource team, such as available resources and political space to change policies. This motivated the choice for a quasi-experimental design because of a stronger engagement with implementation partners and possibility to adapt to changes in context. The limitation of this design is that attribution of causality is more difficult. Furthermore, the limited timeframe of the project may preclude us from fully determining its impact.

The study will allow sharing lessons among the participating countries, and considerably expand the body of knowledge of scale-up for interventions of chronic conditions in health systems in both LMICs and HICs. Other countries can use and adapt the roadmap suitable for their specific context and scaling-up strategy. By such a process, the quality of care and access to care will be optimized according to the needs of each country. By serving as a template for a roll out of integrated care for other chronic conditions and integrated care in times of increasing multimorbidity, the roadmaps to be developed for HT and T2D will be pivotal in placing the concept of scale up of appropriate integrated chronic care on the agenda of stakeholders and governments.

## Supplementary Material

Supplemental Material

## Paper context

There is little knowledge of how to scale-up integrated care for chronic diseases. This study develops and test an innovative three-dimensional scale-up framework to assess progress on population coverage, benefit package, and integration. It provides new possibilities to develop and evaluate scale-up strategies. The development of country-specific scale-up roadmaps, and the cross-lessons gained can be used to tailor scale-up strategies to other health systems with similar typologies.
