# Supplementary material for: Scale-up integrated care for diabetes and hypertension in Cambodia, Slovenia and Belgium (SCUBY): a study design for a quasi-experimental multiple case study
Source: Glob Health Action. 2020 Oct 14;13(1):1824382. doi: 10.1080/16549716.2020.1824382 (PMC7594757; doi:10.1080/16549716.2020.1824382)
Supplement: Supplemental Material [file ZGHA_A_1824382_SM8538.zip › Annex 3 ICP grid_generic.docx]

## Annex 3

## . ICP Implementation Assessment Framework (“Grid”)

**General explanation of the grid:**

Most of the questions in this assessment framework are adapted from the Innovative Care for Chronic Conditions (ICCC) Framework situation assessment (short “grid”)We aim to rate the integrated care package at a particular Unit of Analysis (this is a health care facility in Slovenia and Belgium and an Operational district - including a referral hospital and a number of health centers - in Cambodia). The grid includes sub questions on the five elements of the ICP and additional sub questions on overarching axis that relate to quality control (element 6) .

The elements measure both structure (are the necessary elements present) and process (are processes done consistently and with which level of depth?)

The same grid will be used in each country. (Some of) the questions of the overarching axis (6) will be scores the same for all units of analysis in one particular country, since they are determined by the national context. The country research team can decide to score these questions based upon their knowledge of the national context for all units, and to not include them into the data collection for field researchers.

A translation into the national language will be made by each country team, which will include context-specific clarification of questions.

Instructions for data collection:

Researchers will collect data on the ICP grid for each unit of analysis through multiple sources which they collect during a field visit. At least 2 data collectors will visit one unit of analysis and come to a consensus on scoring comparing their findings. During the field visit they will do:

observations: of the health facility infrastructure, organisation of work, patient flow, interactions of patients with health care workers

informal interviews with health facility managers, health care workers and patients

inspection of documentation at the health facility: management books, patient registries, random patient files check

The grid is filled in immediately after finalising the visit to the unit of analysis. Two researchers will first make an intuitive (“gut feeling”) overall score of the Unit of Analysis (global figure between 1-5) and then they will fill in the detailed grid, each. They will discuss their results and come to one consensus score by the two of them together, also comparing the detailed score with the ‘gut feeling score’.

| **Name of unit tested (group practice, OD, CHC):** | | | | | | | | | | | | | | |
| --- | --- | --- | --- | --- | --- | --- | --- | --- | --- | --- | --- | --- | --- | --- |
| **Name of researchers filling grid:** | | | | | | | | | | | | | | |
| **Date of assessment:** | | | | | | | | | | | | | | |
| **ICP Element** | **Response** | | | | | | | | | | **Method** | **Extra country-specific information** | **ref** | **Instructionsf scoring** |
| Element 1:  **Facility based identification of patients with HT and/or T2DM**  *Facility: place (health center, hospital, clinic, home, headquarter) where care is provided* | No or little implementation | | | | Moderate implementation | | | Almost complete or full implementation | | |  |  |  |  |
| 1.1.a. To what extent, is **screening for DM** performed among patients at a visit? [PROCES] | **0**  Not at all | | **1**  Only on patient’s initiative, not based upon HCW thinking of it = client-based | | **2**  When diabetes symptoms or required by other conditions | **3**  When required by risk factors but not consistently done | | **4**  Consistently done in a group of patients defined by risk factors (almost everyone). | **5**  Everyone who needs to be tested gets tested. | | -Asking health care staff  or/and  -Checking records at the triage if needed (could be in the last 6 months) | In Belgium and Cambodia: screening performed age 40 or over  In Slovenia: age 30 and over  Cambodia: at hospital and health centre.  According to the guidelines and also minimal standard defined in IDF | Yes |  |
| 1.1.b. To what extent, is **screening for HT** performed among patients at a visit? [PROCES] | 0  Not at all | 1  Only on patient’s initiative, not based upon HCW thinking of it = client-based | | | 2  When required by other condition or hypertension symptoms | 3  When required by risk factors but not consistently done | | 4  Consistently done in a group of patients defined by risk factors (almost everyone). | 5  Everyone who needs to be tested gets tested. | | -Asking health care staff  or/and  -Checking records at the triage if needed (could be in the last 6 months) |  | Yes |  |
| 1.2.a. To what extent, are **equipment and materials** necessary for diagnosing patients for **DM** available at the facility?  [STRUCTURE] | 0  Not at all | 1  Available but not functional | | | 2  Partially equipped –some parts not functional | 3  Equipped for a small number of patients | | 4  Fully equipped for almost everyone | 5  Fully equipped for everyone | | -Asking health care staff  -Observation at the facility | It is needed: glucose meter/laboratory (glucose and OGTT)  Mopotsy also considered facility  Laboratory accessible | Yes | Checklist equipment: |
| 1.2.b. To what extent, are **equipment and materials** necessary for diagnosing patients for **HT** available at the facility?  [STRUCTURE] | 0  Not at all | 1  Available but not functional | | | 2  Partially equipped –some parts not functional | 3  Equipped for a small number of patients | | 4  Fully equipped for almost everyone | 5  Fully equipped for everyone | | -Asking health care staff  -Observation at the facility | It is needed: BP meter | Yes | Checklist equipment:  WHO Risk charts  glucometer  Blood Pressure Machine  Stethoscope  Tape measure  cholesterol test strip  urine protein test strip  ketone test strip |
| 1.3.a. To what extent, are health care staff or service providers **competent** to perform **diagnosis for DM** at the facility? [STRUCTURE/EDUCATION] | 0  Not at all | 1  Know but cannot perform properly | | | 2  Perform with guidance from others | 3  Properly perform cannot interpret the results | | 4  Properly perform with limited interpretation of the results | 5  Properly perform with clear interpretation of the results | | -Asking performers or educators (if health care staff is educated about criteria for DM: glucose levels (from 6.1 to 6.9, above 7.0) and about excluding secondary reasons for DM |  | No | Cambodia: Checklist competence:  *know that screening for all who are > 35, who are overweight, who have hypertension, who have (family) history of diabater  know cut-off values for fasting glucose (126 mg/dl)  * know what to if value is high: if no symptoms, repeat; with symptoms make diagnoses[60] |
| 1.3.b. To what extent are health care staff or service providers in charge **competent** to perform **diagnosis for HT** at the facility? [STRUCTURE/EDUCATION] | 0  Not at all | 1  Know but cannot perform properly | | | 2  Perform with guidance from others | 3  Properly performed cannot interpret the results | | 4  Properly performed with limited interpretation of the results | 5  Properly performed with clear interpretation of the results | | -Asking performers or educators (if health care staff is educated about criteria for HT: BP (>140/90) and about excluding secondary reasons for HT |  | No | Cambodia: Checklist competence:  able to explain use of CVD risk chart  -know cut off value of bloodpressure: 140 mm Hg [61] |
| 1.4.a. To what extent is the **follow-up of the patients after the screening, testing and diagnosis of DM** organised? | 0  No follow up | 1  Pt are referred for diagnosis/ therapy but no follow up | | | 2  Follow up only on patient’s initiative | 3  Follow up on if positive/diagnosed and not follow up if negative/high risk | | 4  Follow up if positive and if negative/high risk | 5  Care is organised in such a way there is a planning for every pt and they are called if non- attending | | Asking the nurses, doctors. |  | No |  |
| 1.4.b. To what extent is the **follow-up of the patients after the screening, testing and diagnostic test results** of **HT** organised? | 0  No follow up, it can happen that also no treating of all patients after diagnosisdiagnostics/ therapeutics/ referral options | 1  Pt are referred for diagnosis/ therapy but no follow up | | | 2  If patient is present, follow-up will be performed. | 3  Pt with positive result receives treatment; no follow-up if negative/ high risk | | 4  Pt with positive result receives treatment; follow-up planned if high risk | 5  Care is organised in such a way there is a planning for every pt and they are called if non- attending | | Asking the nurses, doctors. |  | No |  |
|  | | | | | | | | | | | | | | |
| Element 2: **Treatment of DM and HT by primary care providers using standardized protocols**  *Primary care providers: first line of care providers (not including those at hospitals)* | No or little implementation | | | | Moderate implementation | | | Almost complete or full implementation | | |  | - Cambodia: not referring to peer educator |  |  |
| 2.1.a. To what extent, are **written guidelines of care and treatment** accessible to primary care providers for DM? [STRUCTURE] | 0  Not available at all | | | 1  Some guidelines available, but not used in daily practice or difficult to access | 2  Easy accessible guidelines but not recently updated and not encouraged | | 3  Easy accessible guidelines and recently updated or their use is encouraged | 4  Recent updated guidelines available, and their use is encouraged through posters and other educational process etc. | | 5  Recent updated guidelines available and integrated in daily practice through reminders (pop-ups) in electronicmedical record tailored to the patient | -Asking the providers to show guidelines: how easy/difficult the health care workers can show the guidelines when asked to present them.  -Observations at the facility | -All depending on the national guideline: per context identify which guidelines should be present    Encouraged: there are posters and easy available through medical record  Recently updated: in the last 5 years | Yes |  |
| 2.1.b. To what extent, are **written guidelines of care and treatment** accessible to primary care providers for HT? | 0  Not available at all | | | 1  Some guidelines available, but not used in daily practice or difficult to access | 2  Easy accessible guidelines but not recently updated and not encouraged | | 3  Easy accessible guidelines and recently updated or their use is encouraged | 4  Recent updated guidelines available, and their use is encouraged through posters and other educational process etc. | | 5  Recent updated guidelines available and integrated in daily practice through reminders (pop-ups) in electronicmedical record tailored to the patient |  |  | Yes |  |
| 2.2.a. To what extent, are primary care providers in charge **competent to provide treatmen**t for patients with **DM**? [STRUCTURE/EDUCATION]  * professional training = continued professional education | 0  No knowledge at all | 1  Have some non-pharmacological knowledge | | | 2  Have non-pharmacological knowledge and skills | 3  Have detailed knowledge and non pharmacological skills also basic pharmacological knowledge | | 4  Have all detailed knowledge also about pharmacological treatment | 5  Have detailed knowledge and know how to treat complications | | -Asking the providers or educators |  | No | Cambodia: Checklist competence:  -know when to refer (if diagnostic criteria, see under 1.3)  -doctor at NCD should know: step 1 metformin  -step 2 sulfonylurea  step3: insulin 1X  -step4: insuline 1+ at meals  -knows treatment target: HbA&C 7,0%  knows recognition of very high glucose levels (r fasting <180 of postprandial >260) and need for quick action |
| 2.2.b. To what extent, are primary care providers in charge **competent to provide treatmen**t for patients with **HT**? [STRUCTURE/EDUCATION] | 0  No knowledge at all | 1  Have some non-pharmacological knowledge | | | 2  Have non-pharmacological knowledge and skills | 3  Have detailed knowledge and non pharmacological skills also basic pharmacological knowledge | | 4  Have all detailed knowledge also about pharmacological treatment | 5  Have detailed knowledge and know how to treat complications | | -Asking the providers or educators |  | No | Cambodia: Checklist competence, see 6.4 in SOP, pg 20:  -know when to start (repeated 140 if more risk factors, based upon the CVD chart)  -which medication to start: amlodipine and hydrochloorthiazide (12,5 or 25 mg)  - when to refer: if with this med not controlled |
| 2.3.a. To what extent are the **essential medications** for DM available in the primary care setting? [STRUCTURE] | 0  Not at all | | | 1  Only some medications available | 2  Most of the basic medications available, but stock out accure, not indicated in PC | | 3  Most of the basic medications always available and indicated | 4  Also advanced medications available but not indicated in primary care | | 5  Fully accessible to all necessary and all advanced medications (availability and indication) | -Asking the providers and  -Checking drug store (against drugs listed in the national guidelines)  Describe if the problem is because of the indication or stocking out | -All depending on the national guideline | Yes | Cam: see checlist pg 17 [61]: 4-5-6 are for T2D, others are for HT |
| 2.3.b. To what extent, are the **essential medications** for HT available in the primary care setting? [STRUCTURE] | 0  Not at all | | | 1  Only some medications available | 2  Most of the basic medications available, but stock out accure | | 3  Most of the basic medications always available | 4  Also advanced medications available but not indicated in primary care | | 5  Fully accessible to all necessary and all advanced medications | -Asking the providers or  -Checking drug store (against drugs listed in the national guidelines) | -All depending on the national guideline | Yes | Cam: see checlist pg 17 [61] |
| 2.4. To what extent, do primary care providers have necessary **laboratory access**? [STRUCTURE] | 0  Not at all | | | 1  Limited testing items, difficult accessibility | 2  All required testing items, difficult accessibility | | 3  Easy accessibility to limited testing items | 4  Easy accessibility to almost required testing items | | 5  Easy accessibility to all required testing items | -Asking the providers  -Checking laboratory capacity at the facility or in referral | According to national guidelines what are the needed tests (write down in the analysis) | Yes | in hospital lab possible to do:  -HbA1C  lipids  creatinine  potassium  -ALT  -urine analysis (glucose, ketones, protein)  [60] pg 11 |
| 2.5.a. To what extent have primary care providers received **training for treating** DM? | 0  Not at all | | | 1  On the job training or when services started | 2  Part of formal education to obtain certificate needed to do the job | | 3  as in 2 + sporadic extra trainings on the topic | 4  As in 2 + systematically extra trainings on the topic | | 5  As in 2 + systematically extra obligatory trainings on the topic, with innovative methods | -Asking the health care providers  -Checking details of trainings (when, trained by whom, how long, about what) | primary care providers: GP, FP, Rnurse | No |  |
| 2.5.b. To what extent have primary care providers received **training for treating** HT? | 0  Not at all | | | 1  On the job training or when services started | 2  Part of formal education to obtain certificate needed to do the job | | 3  as in 2 + sporadic extra trainings on the topic | 4  As in 2 + systematically extra trainings on the topic | | 5  As in 2 + systematically extra obligatory trainings on the topic, with innovative methods |  | primary care providers: GP, FP, Rnurse | No |  |
| 2.6.a. How comprehensive is **treatment, beyond medication prescription for DM** also including measuring of BMI, waist circumference, BP measurements, cholesterol levels, renal function, screening for complications – foot exam, eye problems, macrovascular disease, depression | 0  Not at all | | | 1  Some elements | 2  Most elements | | 3  All elements not consistently | 4  All elements most of the time | | 5  Systematically all elements | Asking nurses and doctors | According to national guidelines | No |  |
| 2.6.b. How comprehensive is **treatment, beyond medication prescription** **for HT** also including measuring of BMI, BP measurements, palpation of pulsations, cholesterol measurement, ausculation of heart/carotides, screening for complications – urine exam, ECG, CV risk | 0  Not at all | | | 1  Some elements | 2  Most elements | | 3  All elements not consistently | 4  All elements most of the time | | 5  Systematically all elements | Asking nurses and doctors | According to national guidelines | No |  |
| 2.7.a. To what extent are **assessments undertaken in elderly** with DM? (functionality, memory, nutrition) | 0 No questions about functionality memory or nutrition are asked | | | 1 Some questions about functionality memory or nutrition are asked | 2 In specific patients some of these assessments sometimes happen | | 3 In most patients some of the assessments happen | 4 The complete assessment is routinely undertaken: functional, memory and nutrition | | 5 The complete assessment is routinely undertaken: functional, intervention plans are added, eg. Falling | Asking the nurses and doctors |  | No | Part of the guidelines |
| 2.7.b. To what extent are **assessments undertaken in elderly** with HT? (functionality, memory, nutrition) | 0 No questions about functionality memory or nutrition are asked | | | 1 Some questions about functionality memory or nutrition are asked | 2 In specific patients some of these assessments sometimes happen | | 3 In most patients some of the assessments happen | 4 The complete assessment is routinely undertaken: functional, memory and nutrition | | 5 The complete assessment is routinely undertaken: functional, intervention plans are added, eg. Falling | Asking the nurses and doctors |  | No | Part of the guidelines |
| 2.8.a. To what extent are **medication reviews undertaken in elderly** with DM? In order to avoid polypharmacia, hypoglycemia and renal dysfunction. | 0 No medication reviews | | | 1 For some patients GP does this on own initiative | 2 GP does it routinely | | 3 GP does it routinely, sometimes asks advise of farmacist | 4 This is sometimes done in multidisciplinary setting (farmacist) | | 5 This is routinely done in multidisciplinary setting (farmacist) | Asking doctor |  | No |  |
| 2.8.b. To what extent are **medication reviews undertaken in elderly** with HT? In order to avoid polypharmacia, hypoglycemia and renal dysfunction. | 0 No medication reviews | | | 1 For some patients GP does this on own initiative | 2 GP does it routinely | | 3 GP does it routinely, sometimes asks advise of farmacist | 4 This is sometimes done in multidisciplinary setting (farmacist) | | 5 This is routinely done in multidisciplinary setting (farmacist) | Asking doctor |  | No |  |
|  | | | | | | | | | | | | | | |
| Element 3: **Health education and counselling to patients with DM and/or HT by non-physician care providers**  *Non-physician: other than doctors* | No or little implementation | | | | Moderate implementation | | | Almost complete or full implementation | | |  | Non-phys: nurses, peer educations, etc |  |  |
| 3.1.a. To what extent, do patients with DM receive **information on how to reduce health risks** by nurses or community health workers? *(information on chronic disease management and lifestyle support)*  [PROCES] | 0  Not at all | | | 1  Only informal education is given | 2  Within consultation by a non-physician | | 3  Structured individual education by a non-physician is scheduled for every patient | 4  A multidisciplinary team provides structured education to groups of patients | | 5  Group sessions by a multidisciplinary team, the sessions are quality assured (reviewed) and make use of interactive techniques such as video, discussion | -Asking nurses or community health workers | If there is a system that is educating, promoting the education, than there are guidelines how to to the education and also the reports of what was done. | Yes | Cambodia checklist:  -see [60]page 8 |
| 3.1.b. To what extent, do patients with HT receive **information on how to reduce health risks** by nurses or community health workers? *(information on chronic disease management and lifestyle support)*  [PROCES] | 0  Not at all | | | 1  Only informal education is given | 2  Within consultation by a non-physician | | 3  Structured individual education by a non-physician is scheduled for every patient | 4  A multidisciplinary team provides structured education to groups of patients | | 5  Group sessions by a multidisciplinary team, the sessions are quality assured (reviewed) and make use of interactive techniques such as video, discussion | -Asking nurses or community health workers | If there is a system that is educating, promoting the education, than there are guidelines how to to the education and also the reports of what was done. | Yes | Cambodia: checklist see [61] page 19 lifestyle counselling 4 components:  -less salt  -stop smoking  -avoid harmful alcohol  -increase physical act (brisk walking) to at least 30 min per day |
| 3.2.a. To what extent, are patients **informed about the chronic condition** of DM by nurses or community health workers: including the expected course, expected complications, and effective strategies to prevent complications and manage symptoms? *(information on the prognosis)* [PROCES] | 0  Not at all | | | 1  Only informal education is given | 2  Within consultation a non-physician | | 3  Structured individual education by a non-physician is scheduled for every patient | 4  A multidisciplinary team provides structured education to groups of patients | | 5  Group sessions by a multidisciplinary team, the sessions are quality assured (reviewed) and make use of interactive technologies. | -Asking nurses or community health workers  -Checking with patients  -Observation (if possible) |  | Yes |  |
| 3.2.b. To what extent, are patients **informed about the chronic condition** of HT by nurses or community health workers: including the expected course, expected complications, and effective strategies to prevent complications and manage symptoms? *(information on the prognosis)* [PROCES] | 0  Not at all | | | 1  Only informal education is given | 2  Within consultation a non-physician | | 3  Structured individual education by a non-physician is scheduled for every patient | 4  A multidisciplinary team provides structured education to groups of patients | | 5  Group sessions by a multidisciplinary team, the sessions are quality assured (reviewed) and make use of interactive technologies. | -Asking nurses or community health workers |  | Yes |  |
| 3.3.a. To what extent, are nurses or community **health workers trained to provide health education and counselling** to patients with DM? [STRUCTURE/EDUCATION] | 0  Not at all | | | 1  On the job training or when services started | 2  Part of formal education to obtain certificate needed to do the job | | 3  as in 2 + sporadic extra trainings on the topic | 4  As in 2 + systematically extra trainings on the topic | | 5  As in 2 + systematically extra obligatory trainings on the topic, with innovative methods | -Asking nurses or community health workers  -Checking with people at higher level who provide training | Training is done continuously on yearly or 2-yearly basis.   Accreditation: extra training in order to keep their job? | Yes |  |
| 3.3.b. To what extent, are nurses or community **health workers trained to provide health education and counselling** to patients with HT? [STRUCTURE/EDUCATION] | 0  Not at all | | | 1  On the job training or when services started | 2  Part of formal education to obtain certificate needed to do the job | | 3  as in 2 + sporadic extra trainings on the topic | 4  As in 2 + systematically extra trainings on the topic | | 5  As in 2 + systematically extra obligatory trainings on the topic, with innovative methods | -Asking nurses or community health workers  -Checking with people at higher level who provide training | Training is done continuously on yearly or 2-yearly basis.   Accreditation: extra training in order to keep their job? | Yes |  |
| 3.4.a. To what extent, are **health education or counselling materials** accessible to nurses or community health workers for DM? [STRUCTURE] | 0  Not available at all | | | 1  Available (some materials) | 2  All available but not accessible | | 3  Partially accessible to all necessary materials | 4  Fully accessible to almost all necessary materials | | 5  Fully accessible to all necessary materials | -Asking nurses or community health workers  to show it  -Observations | -All (denominator) depending on the national guideline |  |  |
| 3.4.b. To what extent, are **health education or counselling materials** accessible to nurses or community health workers for HT? [STRUCTURE] | 0  Not available at all | | | 1  Available (some materials) | 2  All available but not accessible | | 3  Partially accessible to all necessary materials | 4  Fully accessible to almost all necessary materials | | 5  Fully accessible to all necessary materials | -Asking nurses or community health workers  to show it  -Observations | -All (denominator) depending on the national guideline |  |  |
|  | | | | | | | | | | | | | | |
| Element 4: **Self-management support to patients and their informed caregivers with tools for adherence and monitoring**  *Self-management support: supporting patients to self-manage their conditions (practice and reinforce)* | No or little implementation | | | | Moderate implementation | | | Almost complete or full implementation | | |  |  |  |  |
| 4.1.a. To what extent, are patients offered **self-management training** for DM (for example, to improve adherence to medications, proper nutrition, having self-monitoring tools at home, consistent exercise, tobacco cessation, and maintain other healthy behaviours)? [PROCES] | 0  Not at all | | | 1  Little offer (only one component of the list) to few patients | 2  Limited offer to few patients | | 3  Limited offer to most patients | 4  Offer all the mentioned to most patients | | 5  Offer all the mentioned to every patient | -Asking doctors and nurses |  | Yes | This should not only involve education but be focuses on equiping the patient with skills to solve problems: potential techniques:  -individual or group skill session  -involvement in self-measuring |
| 4.1.b. To what extent, are patients offered **self-management training** for HT (for example, to improve adherence to medications, proper nutrition, having self-monitoring tools at home, consistent exercise, tobacco cessation, and maintain other healthy behaviours)? [PROCES] | 0  Not at all | | | 1  Little offer (only one component of the list) to few patients | 2  Limited offer to few patients | | 3  Limited offer to most patients | 4  Offer all the mentioned to most patients | | 5  Offer all the mentioned to every patient | -Asking doctors and nurses |  | Yes |  |
| 4.2.a. To what extent, do health care staff or community health workers **support patients’ self-management**  efforts **on a continuous basis for DM?** [PROCES] | 0  Not at all | | | 1  Only once when the service starts | 2  In most visits, but no use of telephone/apps | | 3  Once a year via telephone or email | 4  Once per quarter via telephone call or email | | 5  On every visit and supported with commonly used apps | -Asking nurses or community health workers |  | Yes |  |
| 4.2.b. To what extent, do health care staff or community health workers **support patients’ self-management**  efforts **on a continuous basis for HT?** [PROCES] | 0  Not at all | | | 1  Only once when the service starts | 2  In most visits, but no use of telephone/apps | | 3  Once a year via telephone or email | 4  Once per quarter via telephone call or email | | 5  On every visit and supported with commonly used apps | -Asking nurses or community health workers |  | Yes |  |
| 4.3. To what extent, are health care staff or community health workers **competent to perform self-management training?** [STRUCTURE/EDUCATION] | 0  Not at all | | | 1  Know but cannot perform (not confidence or lack of equipment or materials | 2  Can perform with guidance from others | | 3  Can perform limited training lessons | 4  Can perform almost all the training lessons | | 5  Can perform all the training lessons | -Asking the health care staff or community health workers | What do they teach their patients about adherence, self monitoring, nutrition and healthy habits |  |  |
| 4.4.a. To what extent, does the patient have **access to material for self-monitoring for DM**, for instance, glucose meter/ glucose test strips. | **0**  Not exist | | | **1**  Exist in theory but access for patients is not organised | **2**  Access for some patients to all materials needed but refills lack | | **3**  Access for some patients to all materials needed included refills (strips, lancets) | **4**  Well-organised  Access for all patients to all materials needed but refills lack | | **5**  Well-organised   Access for all patients to all materials needed included refills (strips, lancets) | Asking the doctor | Not necessarily all devices and self-management support tools, because also depend on need BUT must be on prescription/for free/available for all |  |  |
| 4.4.b. To what extent, does the patient have **access to material for self-monitoring for HT**, for instance, blood pressure meter | **0**  Not exist | | | **1**  Exist in theory but access for patients is not organised | **2**  Access for some patients to all materials needed but refills lack | | **3**  Access for some patients to all materials needed included refills (strips, lancets) | **4**  Well-organised  Access for all patients to all materials needed but refills lack | | **5**  Well-organised   Access for all patients to all materials needed included refills (bateries) | Asking the doctor s/nurses |  |  |  |
| 4.5.a. To what extent are **informal caregivers/non medical** involved in the self-management process for DM?  (i.e. family, social worker, community workers, organisations - it must be informal not part of health care system) | **0**  Not exist | | | **1**  Occasionally involved but no health knowledge | **2**  Occasionally involved but limited health knowledge | | **3**  Fully involved with limited health knowledge | **4**  Fully involved with full knowledge but haven’t received any supporting materials | | **5**  Fully involved with full knowledge and supporting materials | Asking the doctors/nurses | Informal/non medical caregiver: community worker (volunteer health support group) and peer educator |  |  |
| 4.5.b. To what extent are **informal caregivers/non medical** involved in the self-management process for HT? | **0**  Not exist | | | **1**  Occasionally involved but no health knowledge | **2**  Occasionally involved but limited health knowledge | | **3**  Fully involved with limited health knowledge | **4**  Fully involved with full knowledge but haven’t received any supporting materials | | **5**  Fully involved with full knowledge and supporting materials | Asking the doctors/nurses | Informal/non medical caregiver: community worker (volunteer health support group) and peer educator |  |  |
| 4.6. Are the **concerns of patients and families** addressed? | 0  Not at all | | | 1 Is not consistently done | 2 Is provided for specific patients and families through referral | | 3 Is provided for specific patients and families in primary care | 4 Is encouraged, and peer support, groups and mentoring programs are available | | 5 Is an integral part of primary care and includes systematic assessment and routine involvement in peer support, groups or mentoring programs | Ask doctor and nurse | Peer support: group of patients that talk amongst each other | YES ACIC |  |
| 4.7. Are **patient treatment plans** used, are they agreed with patients, reviewed and written down? | 0 patient plans are not expected | | | 1 patient treatment plans only sometimes written down just | 2 pt treatment plans are achieved through a standardized approach for majority of patients | | 3 are established collaboratively with patient and include clinical goals | 4 are established collaboratively with patient and include clinical goals as well as self management | | 5 are established collaboratively with patient and include clinical goals as well as self management. Follow-up occurs and guides care | Ask the doctors and nurse | Yes acic |  |  |
|  | | | | | | | | | | | | | | |
| Element 5: **Structured collaboration between health care workers, community actors, and patients and caregivers** | No or little implementation | | | | Moderate implementation | | | Almost complete or full implementation | | |  |  |  |  |
| 5.1.a. To what extent, is there an identified **“care coordinator”** who serves as the overseer and director of a patient’s care, ensuring that efforts of all involved health care workers, community actors, and patients and caregivers are integrated and coordinated for DM? [STRUCTURE] | 0  Not exist | | | 1  Exist but not active | 2  Exist and active only when triggered | | 3  Exist and active occasionally | 4  Exist and active but not structured | | 5  Exist and active and structured | -Asking the health care staff  -Checking in the community |  | Yes |  |
| 5.1.b. To what extent, is there an identified **“care coordinator”** who serves as the overseer and director of a patient’s care, ensuring that efforts of all involved health care workers, community actors, and patients and caregivers are integrated and coordinated for HT? [STRUCTURE] | 0  Not exist | | | 1  Exist but not active | 2  Exist and active only when triggered | | 3  Exist and active occasionally | 4  Exist and active but not structured | | 5  Exist and active and structured | -Asking the health care staff  -Checking in the community |  | Yes |  |
| 5.2.a. To what extent, do the **health care organization and the community** have **complementary functions**, that is, community organizations fill gaps in services that are not provided in formal health care for DM? [STRUCTURE] | 0  Not at all | | | 1  Community effort exists but not relevant to the gap | 2  Community effort exists and relevant but unable to fill the gaps | | 3  Community exists and filling limited gaps | 4  Community exists and almost filling the gaps | | 5  Community exists and filling all the gaps | -Asking the health care staff and patients  -Checking in the community | Gaps of the continuity of care  Local system | Yes |  |
| 5.2.b. To what extent, do the **health care organization and the community** have **complementary functions**, that is, community organizations fill gaps in services that are not provided in formal health care for HT [STRUCTURE] | 0  Not at all | | | 1  Community effort exists but not relevant to the gap | 2  Community effort exists and relevant but unable to fill the gaps | | 3  Community exists and filling limited gaps | 4  Community exists and almost filling the gaps | | 5  Community exists and filling all the gaps | -Asking the health care staff and patients  -Checking in the community | Gaps of the continuity of care | Yes |  |
| 5.3.a. To what extent, are **referral practices** systematically organisedfor DM? [STRUCTURE] | 0  Not at all | | | 1  Limited Referral and only one direction | 2  Referral and only one direction | | 3  Referral organisedinformally for two directions | 4  Limited Referral organisedsystematically for two directions | | 5  Referral structured systematically for two directions | -Asking the health care staff  Referral back: report and what does primary care doctor needs to do | Explain of grading 3) moderate implementation could be systematic referral in two ways, but the full implementation could be teams that communicate with each other about a patient that goes beyond a referral letter and that they also meet each others sometimes | Yes where? |  |
| 5.3.b. To what extent, are **referral practices** systematically organisedfor HT? [STRUCTURE] | 0  Not at all | | | 1  Limited Referral and only one direction | 2  Referral and only one direction | | 3  Referral organisedinformally for two directions | 4  Limited Referral organisedsystematically for two directions | | 5  Referral organisedsystematically for two directions | -Asking the health care staff  Referral back: report and what does primary care doctor needs to do | Explain of grading 3) moderate implementation could be systematic referral in two ways, but the full implementation could be teams that communicate with each other about a patient that goes beyond a referral letter and that they also meet each others sometimes | Yes |  |
| 5.4.a. To what extent does **cooperation between health care workers and patients** occur for DM? | 0  No cooperation | | | 1  Little cooperation without regular discussions | 2  Moderate cooperation within the team with regular discussions | | 3  Full cooperation within teams, but not across | 4  Cooperation within teams and across teams to some external team | | 5  Multi-disciplinary cooperation across all levels | Multidisciplinary: doctor, nurse and other health care specialists | Eg. effective communication abilities to promote information exchange, open questioning, and shared decision-making with patients? | Yes |  |
| 5.4.b. To what extent does **cooperation between health care workers and patients** occur for HT? | 0  No cooperation | | | 1  Little cooperation without regular discussions | 2  Moderate cooperation within the team with regular discussions | | 3  Full cooperation within teams, but not across | 4  Cooperation within teams and across teams to some external team | | 5  Multi-disciplinary cooperation across all levels | Multidisciplinary: doctor, nurse and other health care specialists | Eg. effective communication abilities to promote information exchange, open questioning, and shared decision-making with patients? | Yes |  |
| 5.5.a. To what extent is the traditional **hierarchy flattened** and moved away from physician dominated models for DM? | 0 Specialists are dominating, also in first-line | | | 1 For some patients specialists are central, for other general practitioners | 2 General practitioners are central, other HCW play minor role | | 3 General practitioners are central, other HCW play big role | 4 There is a multidisciplinary team and everyone is considered equal | | 5 HCW with special training in chronic care take the lead. |  |  | Yes |  |
| 5.5.b. To what extent is the traditional hierarchy flattened and moved away from physician dominated models for HT? | 0 Specialists are dominating, also in first-line | | | 1 For some patients specialists are central, for other general practitioners | 2 General practitioners are central, other HCW play minor role | | 3 General practitioners are central, other HCW play big role | 4 There is a multidisciplinary team and everyone is considered equal | | 5 HCW with special training in chronic care take the lead. |  |  | Yes |  |
|  | | | | | | | | | | | | | | |
| Element 6: **Questions regarding organisation of care, delivery system design and clinical information systems** | No or little implementation | | | | Moderate implementation | | | Almost complete or full implementation | | |  |  |  |  |
| 6.1 To what extent are ongoing **quality improvement** routine activities among health care workers organised? | 0  No quality improvement | | | 1  New rules to improve quality are sometimes set from management | 2  When a problem pops up a quality improvement activity is sometimes undertaken | | 3  When a problem pops up a quality improvement activity is often undertaken | 4  Is a routinely process but results from previous round are often not taken into account | | 5  Is a routinely process and results from previous round are taken into account |  |  | Yes |  |
| 6.2 To what extent do **information systems** gather and organise data about epidemiology, treatment, and health care outcomes? | 0  There is no registry | | | 1  There is a registry but is not used for treatment purpose | 2  The registry includes name, diagnosis, contact info and date of last contact | | 3  The registry includes name, diagnosis, contact info, date of last contact, treatment and outcomes | 4  The registry allows queries to sort subpopulations by clinical priorities | | 5  The registry is tied to guidelines which provide prompts and reminders about needed services | For specific population in each site |  | yes |  |
| 6.3 To what extent is **information about relevant subgroups** of patients needing services available? | 0  is not available | | | 1  can not be obtained by primary care provider | 2  can only be obtained with special efforts or additional programming and is incomplete | | 3  can only be obtained with special efforts or additional programming | 4  can be obtained upon request but is not routinely available | | 5  is provided routinely to providers to help them deliver planned care and is used for this purpose |  | Stratification - how many people have special needs  SLO: older than 65, multimorbid  BE:  CAMB: | yes |  |
| 6.4 To what extent do information systems serve a **reminder function** for patient specific prevention and follow-up services (e.g. to identify patients’ needs, to follow-up and plan care, to monitor responses to treatment, and to assess health outcomes)? | 0  No Information system | | | 1  No reminder function | 2  Reminders include general notification of the existence of a chronic illness, but does not describe needed services at time of encounter | | 3  Reminders describe needed services at time of encounter, based on general guideline, not patient-specific | 4  Includes specific information for the patient at the time of individual patient encounters | | 5  Includes specific information for the team about adherence to patient care plan at the time of individual patient encounters |  |  | yes |  |
| 6.5 To what extent is **feedback about the performance provided to the team and its members**? | 0  Not available | | | 1  Non-specific to the team | 2  Infrequent intervals and not delivered to the team (teams if they want they search for the information) | | 3  Frequent intervals but not specific for the team and impersonally delivered (just common reports) | 4  Occurs at frequent enough intervals to monitor performance and is specific to the team’s population | | 5  Timely, specific to the team, routine and personally deliveredby a respected opinion leader |  | CHC manager feedback, how you are performing.  Comparison between the different CHC in the country (Lab tests, prescriptions) | yes |  |
| 6.6 To what extent is an **appointment system** with planned visits used? | 0  No appointment system | | | 1  Used to schedule acute care visits, follow-up and preventive visits | 2  Appointment system assures scheduled follow-up with chronically ill patients, but some pts escape the system | | 3  Appointment system assures scheduled follow-up with chronically ill patients | 4  Appointment system are flexible and can accommodate innovations such as customized visit length or group visits | | 5  Appointment system includes organization of care that facilitates the patient seeing multiple providers in a single visit. |  | Comment what is actually going on | Yes |  |
