# Supplementary material for: Scale-up integrated care for diabetes and hypertension in Cambodia, Slovenia and Belgium (SCUBY): a study design for a quasi-experimental multiple case study
Source: Glob Health Action. 2020 Oct 14;13(1):1824382. doi: 10.1080/16549716.2020.1824382 (PMC7594757; doi:10.1080/16549716.2020.1824382)
Supplement: Supplemental Material [file ZGHA_A_1824382_SM8538.zip › Annex 4 Questions Focus Groups_20190513_clean.docx]

**Topic guide for FOCUS GROUPS**

This topic guide is a tool for step 1.3 of the protocol: organisational context.

**The aim of the focus group discussion is:**

- to complement data collection needed to understand the organisational context. (step 1.3)
- to completement the filling of the ICP immplementation grid (step 1.2)
- to complement infrmation needed for step 1.4 (cost provider perspective) and step 2.3 (cost and barriers patient perspecive)
- to understand the *different* perspectives of key stakeholders, namely frontline imlpementers and beneficiaris/patients, on the current process of ICP delivery and scale-up

**Methods of respondent selection and data collection**

In a context where multidisciplinary teams are functional, we will have one FGD with medi cal professionals: medical doctor, other health worker and community health worker together (focus group discussion with a team: the teams can include everyone that is systematically involved in the interaction with the patient. The health professionals could be joined in one group, if better adapted to teh context. In Slovenia, this is the group of prevention team (CKZ), in Cambodia, this can include community workers and family (informal caregivers). Patients will be selected from the target population (Slovenia and Belgium older that 65 and/or having multimborbidity). Exclusion criteria are: not being able to hear or talk clearly.

This tool includes the generic topic guide for the focus group discussions. Group-specific questions are listed for patients and health care workers/teams. General demographic questions on gender, age, multimorbidity/diseases, living environment and the type of ICP delivery model, will be collected for, to understand the variety of respondent in the group.

**MAIN TOPICS**

First a short introduction of SCUBY, aim of focus group discussion, informed consent

**Topic Guide for Health care professionals**

Introduction

Opinion on current process

- Which adaptations were made at the implementation of the integrated care package at the health care facility and at other organisations involved?
- Which external organisations were involved to support implementation?
- How were health workers, other actors and patients prepared for implementation?
- What was the role of local and central health authorities in the implementation?
- In your opinion, what is good in the current implementation of the integrated care for people with diabetes and hypertension?

Reach of vulnerable populations

- How are vulnerable populations identified? How well does the ICP reach vulnerable people? (Slo/Bel)

Facilitators

What are important factors that facilitate the current way of working?° What are further options for improving management of people with T2D and/or hypertension? (Slo/Bel) Especially when you think about vulnerable people?

° How do you see the directions of possible extension of care /scaling up and facilitators in doing that?

° Who are potential people/approaches that can be added to the existing care. What view do you have on informal caregivers, patients as teachers and other informal staff?

- How do you see a role of patients? Of organisations in the community? Of informal care givers?

**Obstacels**

° What are your personal experiences / what kind of obstacles (in screening, testing, treatment) have you considered in the ICP process (screening, testing, diagnosing, retained in care, follow up, self management, different levels cooperation) for (vulnerable) patients with HY/T2D at the primary level?

° What are the problems of people who dont come regularly for care? What are current actions you take? What ware other options?

° Where are the obstacles that would prevent expansion or change in the process?

**Topic Guide for Patients**

Introduction

Opinion on current process

- What is good in the existing process
- Where are you seeking / gaining the most knowledge and support / what kind of self management does it help you/do you use?
- What view do you have on informal caregivers, patients as teachers and other informal staff? Who are potential people/approaches that can be added to the existing care?
- In what way do you see the care to develop in order to give you the greatest support and to gain the most out of it?

Barriers: financial and non-financial

- Where do you usually go for care for your diabetes/hypertension care?
- Are there other people you go to for your diabete/hypertension apart from your doctor?
- Are there people in your environment who help you in the management of your diabtees/Hypertension?
- Are there organisations outside of the hospital that are of help in the management of your disease?
- Do you experience problems/obstacles in getting care? Can you elaborate?
- Direct medical cost
- other costs than direct medical care Think of transport, of diet, etc
- Time needed for health care visits
- Changes of this over time
- Are there some services available which could be useful but unaffordable or difficult access? Do you know people who were able to get access to such services? How do they manage?

**OBSTACLES IN SCALING UP**

- What do you feel it is not so good in the process that it can be changed?
- Where are the obstacles that would prevent expansion or change in the process?
