# Supplementary material for: Scale-up integrated care for diabetes and hypertension in Cambodia, Slovenia and Belgium (SCUBY): a study design for a quasi-experimental multiple case study
Source: Glob Health Action. 2020 Oct 14;13(1):1824382. doi: 10.1080/16549716.2020.1824382 (PMC7594757; doi:10.1080/16549716.2020.1824382)
Supplement: Supplemental Material [file ZGHA_A_1824382_SM8538.zip › Annex 5 Costing tool Cam_version fin.docx]

### Annex 5. Rapid facility-based survey costing Cambodia. Supply-side costing of the **existing** integrated care package

This tool will be used to assess cost for the ICP from the provider perspective. It will be filled in by ta researcher, based upon observations, document review and key informant interviews during a 1 week field visits. The health facilities are selected following the sampling grid for Cambodia (Annex 6), so 3 health facilities in 5 Operational Districts.

This tool summarizes cost categories by the five intervention components. Cost data will depend on the existing activities related to the five components. If it is not possible to assign cost to a specific ICP category, it can be put under a column “overall”.

| No. | Cost items | Early case detection/ notification | Treatment in primary care services | Health education and counselling | Self-management support | Collaboration between care givers |
| --- | --- | --- | --- | --- | --- | --- |
| 1 | Cost for staff training/capacity building |  |  |  |  |  |
| 2 | Cost of equipment (depreciation & maintenance) |  |  |  |  |  |
| 3 | Personnel/Labor cost |  |  |  |  |  |
| 4 | Travel cost |  |  |  |  |  |
| 5 | Medicines |  |  |  |  |  |
| 6 | Materials/tests |  |  |  |  |  |
| 7 | Administration/overhead cost |  |  |  |  |  |
| TOTAL COST | |  |  |  |  |  |
