# Supplementary material for: Scale-up integrated care for diabetes and hypertension in Cambodia, Slovenia and Belgium (SCUBY): a study design for a quasi-experimental multiple case study
Source: Glob Health Action. 2020 Oct 14;13(1):1824382. doi: 10.1080/16549716.2020.1824382 (PMC7594757; doi:10.1080/16549716.2020.1824382)
Supplement: Supplemental Material [file ZGHA_A_1824382_SM8538.zip › Annex 6 pt cost.docx]

## Annex 6. Costing and barriers patient perspective

These are guiding questions that will be adapted to the relevance in each country

**Health care seeking**

(Cam) Where do you (or your family members) usually go for care when you (or your family members) get ill or injured? A list of types of facilities/providers as options for answers;

For those with known T2D and/or HT, where did you first get to know your T2D and/or HT status (detected/diagnosed)? A list of types of facilities/providers as options for answers, similar to the above question;

Have you been seeking any care/treatment for your T2D and/or HT? Yes/No;

Where do you go for your routine treatment for diabetes?

Where do you go for your routine treatment for hypertension?

How many times per year do you go to see a medical specialist doctor in the hospital for your diabetes treatment?

How many times per year do you go to see a medical specialist doctor in the hospital for your hypertension treatment?

How much did you spend to get to know your T2D and/or HT status (detected/diagnosed)? The expenditure includes service fee (for the facility/provider and test), transportation cost, and food

Have you ever been admitted in the hospital last year? Yes/No;

Was the hospitalization related to (for the complications of) your T2D and/or HT? Yes/No/Don’t know

Do you also go to other people/organisations for your health? Probing questions (country specific: Cambodia: complementary/herbal medicine – Belgium: alternative health care providers, dietician, psychologist – Slovenia: diabetes association, health promotion center)

**Health care expenditure**

How much did you spend to get health care last month/last year? The expenditure includes service fee (for the facility/provider), transportation cost, and food;

How much did you spend on cost that were not reimburse by the health insurance last year/month?

If you got admitted in the hospital, how much did you spend for such hospitalization? If more than once, multiply the figure to get all expenditure.

**Barriers to access to care**

Have there been times that you have not go to the health provider for your diabetes or hypertension while you actually needed or wanted to go?

If so, what were the reasons that you did not go? (optional: A list of major reasons for delaying or foregoing care, including the perception of unnecessary, as options for answers (multiple choices).

**Background questions, needed to understand variation**

What is your age?

Do you have diabetes? Yes/no

Do you have hypertension? Yes/no

Are you treated for any other chronic disease? Make list to choose from

Kidney problem

Cardiac disease

Chronic lung disease

Neurologic disease

Depression

Other mental illness

Do you have entitlements for reduced fees (in Belgium: “verhoogde tegemoetkoming”, in Cambodia “health equity fund”, in Slovenia….)

**DIARY FOR PATIENT DAILY NOTES (Cambodia)**

Patient/Household ID: ______________________­­­­__________­­­­­

Address: ________________________________

Registered to/In the catchment area of HC/RH/OD: _______________________________

The starting date of the diary: ________/________/______________

**INSTRUCTIONS FOR COMPLETION OF THE DIARY**

This diary should be completed DAILY by the patient or spouse or any adult member of the household, who can read and write

If s/he takes medicines available at home or bought from drug seller/pharmacy by the family, going to see a medical person for advice/diagnosis/treatment, admitted to a clinic or hospital and the amount of money spent for the medicines, fees, transportation, food..., please write down all the detailed information as indicated in the example below

If there is no care seeking, please write NO

This diary will be reviewed and collected by the research team every month. During the review, diary keeper could be asked for clarifications or more information by the research team

Patient who has spent time to appropriately completing this diary will be compensated by the research team with US$1/month.

We thank you in advance for spending your valuable time to fill this diary.

All the information will be kept confidential

| Date and time | No | Illness/injury: symptoms, diagnosis and condition or reasons for seeking care | Medicines taken: name/type and source | Medical consultation: name of medical person/ facility, advices/care | Hospitalization: name of facility, advices/care | Money spent for: medicines, formal and informal fees, transportation, food... |
| --- | --- | --- | --- | --- | --- | --- |
| **DAY 01 (until 30)** | | | | | | |
|  |  | ________________  ________________ | ________________  ________________ | ________________  ________________ | ________________  ________________ | ________________  ________________ |
